# Hyperspectral Imaging for Enhanced Skin Cancer Classification Using Machine Learning

**DOI:** 10.3390/bioengineering12070755

**Published:** 2025-07-11

**Authors:** Teng-Li Lin, Arvind Mukundan, Riya Karmakar, Praveen Avala, Wen-Yen Chang, Hsiang-Chen Wang

**Affiliations:** 1 Department of Dermatology, Dalin Tzu Chi Hospital, No. 2, Min-Sheng Rd., Dalin Town, Chiayi 62247, Taiwan; tanglilin1121@hotmail.com; 2 Department of Mechanical Engineering, National Chung Cheng University, 168, University Rd., Min Hsiung, Chiayi 62102, Taiwan; d09420003@ccu.edu.tw (A.M.); karmakarriya345@gmail.com (R.K.); 3 Department of Biomedical Imaging, Chennai Institute of Technology, Sarathy Nagar, Chennai 600069, Tamil Nadu, India; 4 Department of Computer Science Engineering, Vel Tech Rangarajan Dr. Sagunthala R&D Institute of Science and Technology, No. 42, Avadi-Vel Tech Road Vel Nagar, Avadi, Chennai 600062, Tamil Nadu, India; vtu17283@veltech.edu.in; 5 Department of General Surgery, Kaohsiung Armed Forces General Hospital, 2, Zhongzheng 1st. Rd., Kaohsiung City 80284, Taiwan; 6 Technology Development, Hitspectra Intelligent Technology Co., Ltd., 4F., No. 2, Fuxing 4th Rd., Qianzhen Dist., Kaohsiung City 80661, Taiwan

**Keywords:** skin cancer, hyperspectral imaging, spectrum-aided vision enhancer, convolutional neural network, yolo, random forest, narrow-band imaging, band selection

## Abstract

**Objective:** The classification of skin cancer is very helpful in its early diagnosis and treatment, considering the complexity involved in differentiating AK from BCC and SK. These conditions are generally not easily detectable due to their comparable clinical presentations. **Method:** This paper presents a new approach to hyperspectral imaging for enhancing the visualization of skin lesions called the Spectrum-Aided Vision Enhancer (SAVE), which has the ability to convert any RGB image into a narrow-band image (NBI) by combining hyperspectral imaging (HSI) to increase the contrast of the area of the cancerous lesions when compared with the normal tissue, thereby increasing the accuracy of classification. The current study investigates the use of ten different machine learning algorithms for the purpose of classification of AK, BCC, and SK, including convolutional neural network (CNN), random forest (RF), you only look once (YOLO) version 8, support vector machine (SVM), ResNet50, MobileNetV2, Logistic Regression, SVM with stochastic gradient descent (SGD) Classifier, SVM with logarithmic (LOG) Classifier and SVM- Polynomial Classifier, in assessing the capability of the system to differentiate AK from BCC and SK with heightened accuracy. **Results:** The results demonstrated that SAVE enhanced classification performance and increased its accuracy, sensitivity, and specificity compared to a traditional RGB imaging approach. **Conclusions:** This advanced method offers dermatologists a tool for early and accurate diagnosis, reducing the likelihood of misclassification and improving patient outcomes.

## 1. Introduction

Given that the skin is the body’s biggest organ, it is reasonable to regard skin cancer as the most prevalent kind of cancer in humans [1]. As of 2020, skin malignancy ranks as the fifth most prevalent cancer globally, according to the World Health Organization [2]. Skin cancer is primarily categorized into two principal types: malignant melanoma (MM) and non-melanoma skin cancer (NMSC). Despite the incidence rate of non-melanoma skin cancer (NMSC) being significantly higher than that of malignant melanoma, the death rate associated with melanoma is greater [3,4]. The primary forms of non-melanoma skin cancer (NMSC) include basal cell carcinoma (BCC), actinic keratosis (AK), and seborrheic keratosis (SK) [5]. Inability to produce rapid reports from medical imaging postpones patient care, and misdiagnosis may result in detrimental situations that could lead to patient mortality [6]. In the diverse realm of machine learning applications within healthcare, medical image classification, especially in dermatology, has significantly progressed in recent years, encompassing the identification of skin malignancies using dermoscopic or macroscopic images [7].

Monika et al. examined a methodology utilizing MSVM classification, employing two efficient techniques, ABCD and MSVM, for feature extraction, which attained an accuracy of 96.25% in classifying eight distinct forms of skin malignancies [8]. Javaid et al. developed an innovative technique for skin cancer classification via machine learning and image processing, applied with the ISIC dataset for malignant and benign skin cancers, including support vector machines (SVMs), quadratic discriminant analysis, and random forest algorithms [9]. Murugan et al. suggested a skin cancer detection system utilizing image processing to identify SK, BCC, and melanoma, presenting an enhanced diagnostic method compared to the conventional biopsy technique [10]. Vuran et al. developed a multi-class, fast, and reliable autonomous disease diagnosis model using transformer-based deep learning architectures and skin lesion images, including for Mpox disease [11]. Nevertheless, the majority of these research studies have exclusively utilized RGB pictures for the identification and categorization of skin lesions. The integration of hyperspectral imaging (HSI) with narrow-band imaging (NBI) enhances the contrast between malignant and normal tissues, hence improving the precision and recall of detection.

NBI is a method that use blue and green light to accentuate mucosal and submucosal blood vessels, facilitating improved identification of (pre)malignant lesions exhibiting abnormal blood vessel patterns [12]. The NBI filter confines the light spectrum to two narrow bands: blue at 415 nm and green at 540 nm [13]. The 415 nm wavelength improves the imaging of superficial veins and mucosal structures, but the 540 nm wavelength promotes the vision of deeper structures, including sub-epithelial capillaries [14]. Research on light interaction with biological tissues has demonstrated that longer wavelengths penetrate tissues more deeply [15]. Zwakenberg et al. demonstrated that narrow-band imaging (NBI) enhances the visibility of tumor extension and the precision of T staging [16]. Russo et al. found that NBI exhibited enhanced illness sensitivity and specificity relative to WLI, along with a superior overall hierarchical summary receiver operating characteristic [17]. Staudenmann et al. found that NBI is a potential method for identifying benign laryngeal lesions due to its optical characteristics [18].

HSI is an innovative technology that utilizes the entire electromagnetic spectrum rather than the conventional three bands (red, green, and blue), thus extracting far more information from each pixel [19]. HSI acquires two-dimensional (2-D) spectral and one-dimensional (1-D) spatial data, resulting in a three-dimensional (3-D) hypercube. The hypercube facilitates the differentiation or categorization of sample kinds, a capability unattainable by traditional color imaging techniques [20]. NBI, in conjunction with HSI, has been employed in several applications, including remote sensing [21], agriculture [22], counterfeit detection [23], and for numerous biomedical imaging techniques, such as the detection of esophageal cancer [24], head and neck cancer, skin cancer [25] and prostate cancer [26]. Nevertheless, the band selection approach has not been extensively employed in the identification or categorization of skin cancer.

Therefore, in this study, HSI conversion technology that has the ability to convert the RGB images into an NBI image known as SAVE has been combined with a computer-aided diagnosis (CAD) system to classify skin cancer, including BCC, SK and AK. The RGB images have been converted to SAVE images in specific bands that increase the contrast of the cancerous tissues and the RGB dataset and SAVE dataset have been trained with multiple machine learning models, including CNN, RF, YOLOv8, SVM, ResNet50, MobileNetV2, Logistic Regression, SVM- SGD Classifier, SVM- LOG Classifier and SVM- Polynomial Classifier. Following the Section 1, the Section 2 gives a detailed description of the dataset, SAVE algorithms, and machine learning algorithm, while the Section 3 provides the results obtained in this study. The Section 4 discusses the limitations and the future scope of this study, while the Section 5 gives the conclusion of this study. This study’s principal contributions are outlined as follows:

- We introduce the innovative SAVE algorithm, which utilizes HSI and band selection methods to convert ordinary RGB images into NBI like with improved lesion contrast.
- We assess and contrast the efficacy of ten distinct machine learning algorithms—including CNN, YOLOv8, ResNet50, and several SVM classifiers—in the precise classification of AK, BCC, and SK.
- We illustrate that SAVE-enhanced imaging markedly enhances classification accuracy, sensitivity, and specificity compared to conventional RGB imaging, with the CNN attaining the greatest accuracy of 98%. Our methodology offers a pragmatic instrument that aids dermatologists in the early and accurate identification of skin cancer, thereby diminishing misdiagnosis and enhancing patient outcomes.
- These contributions enhance the forefront of dermatological imaging and machine learning-driven cancer categorization, presenting intriguing avenues for future clinical applications.

## 2. Materials and Methods

### 2.1. Dataset

**Figure 1:**
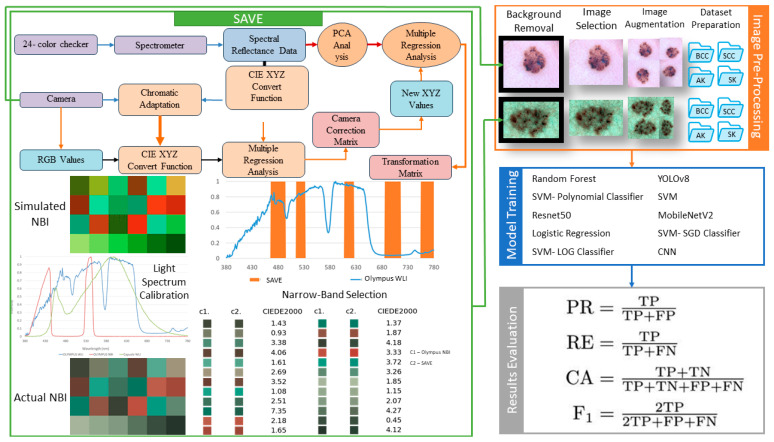
Workflow of the SAVE system, illustrating the process from color calibration and spectral data acquisition, through simulated NBI generation and image pre-processing, to dataset preparation, machine learning model training, and evaluation using key performance metrics.

This study employed 954 images to analyze four unique classifications of skin cancer: AK with 288 images, BCC with 338 images, and SK with 328 images, all obtained from the International Skin Imaging Collaboration (ISIC) website (https://www.isic-archive.com/) (accessed on 12 February 2025). All images were standardized to a resolution of 640 × 640 pixels during preprocessing to alleviate potential issues, such as insufficient computer memory, and to ensure format uniformity. Image annotation was conducted using the Roboflow software platform. An XML file was generated and later transformed into a text file. The annotated dataset was transformed into SAVE images using the SAVE NBI transformation approach during the inquiry. As a result, two datasets were acquired utilizing WLI and SAVE images. The distribution of images within each diagnostic group demonstrates a notable imbalance, perhaps resulting in bias in the model’s effectiveness. The model utilizes a balanced approach to mitigate the impact of any one modality on the outcomes, notwithstanding the variations in image quantity across modalities. This entails utilizing normalization and augmentation methods to alleviate the effects of modality-specific characteristics. Our model’s methodology incorporates diverse images from several modalities, augmenting its capacity to generalize across various input sources. The use of many imaging modalities in the model’s training data is advantageous rather than restrictive, since it allows the model to proficiently navigate real-world situations. The performance parameters, encompassing accuracy, recall, and mean average precision (mAP), are obtained after thorough validation and testing across all modalities. This guarantees the model’s performance remains constant and dependable, regardless of the type of picture. Our study primarily concentrates on detecting fundamental illness patterns rather than surface visual attributes. The overall flowchart is shown in Figure 1.

### 2.2. Spectrum-Aided Vision Enhancer

This study established a VIS-HSI conversion mechanism that can convert an RGB picture obtained from an endoscope into an HSI image and a simulated NBI image referred to as SAVE, as illustrated in Figure 1. Initially, it is essential to determine the connection between the RGB picture and the spectrometer for different colors. The Macbeth Color Checker (X-Rite Classic) is designated as the color set for calibration purposes. This tool consists of 24 squares showcasing several color samples commonly seen in nature, including shades such as red, green, blue, cyan, magenta, and yellow, along with six tones of gray. X-Rite has been a favored choice for color calibration in recent years. The camera was primarily utilized to capture images that faithfully depicted the colors of the X-Rite board, referred to as the target. The picture of 24 colors was transformed into the CIE 1931 XYZ color space. The endoscope captured the image and stored it in JPEG format with the standard RGB (sRGB) color scheme. The R, G, and B values of the sRGB color space, spanning from 0 to 255, were first transformed to a more restricted range of 0 to 1. Thereafter, the Gamma function was utilized to transform the reduced sRGB values into linearized RGB values. A translation matrix was utilized to transform the linearized RGB values into the CIE 1931 color space, illustrating the numerical correlation between the wavelengths in the visible spectrum and the colors observed in nature. Conversely, images captured with an endoscope may be affected by non-linear response, dark current, improper color separation, or color distortion. Consequently, Equation (1), including a matrix of variables, was utilized. Equation (2) was employed to compute the revised X, Y, and Z values (XYZ_correct_) subsequent to error correction.

(1) $$\left[C\right]=\left[{XYZ}_{Spectrum}\right]\times pinv(\left[V\right])$$

(2) $$\left[{XYZ}_{Correct}\right]=\left[C\right]\times [V]$$

The spectrometer employed in this study was the Ocean Optics QE65000 (Orlando, FL, USA), which was compatible with the X-Rite board. This research acquired a reflectance spectrum of a 24-color patch with this spectrometer. The brightness ratio was obtained from the Y value of the XYZ color gamut space, as this parameter directly correlates with brightness. The reflectance spectrum data were transformed into XYZ values (XYZ_Spectrum_) and subsequently normalized within the XYZ color gamut space. The correction coefficient matrix *C* was obtained by multiple regression, especially utilizing Equation (3). The reflectance spectrum data (R_spectrum_) were employed to compute the transformation matrix (*M*) for the colors included in the X-Rite board. Principal component analysis (PCA) was performed on the R_spectrum_ dataset to identify the six most important principal components (PCs) and their associated eigenvectors. The six personal PCs accounted for 99.64% of the data. The average root mean square error (RMSE) of the 24 target colors between XYZ_correct_ and XYZ_Spectrum_ was 0.19, signifying that the discrepancy is negligible. A multiple regression analysis was subsequently performed on variable *M* to investigate its link with the six major main components. Six PCs were employed to do a multivariate regression study of XYZ_correct_. This study meticulously chose the variable V_color_ for its capacity to incorporate all potential combinations of the X, Y, and Z values.

[M] = [Score] × pinv([V_Color_]) (3)

[S_Spectrum_]_(380~780 nm)_ = [EV][M][V_Color_] (4)

The analog spectrum (S_Spectrum_) was derived from XYZ_correct_ utilizing Equation (4). Subsequently, S_Spectrum_ was evaluated against R_spectrum_. The average RMSE of the 24 color blocks was 0.056, and the mean color deviation between the obtained analog spectrum and the reflectance spectrum generated by the spectrometer was 0.75. This observation indicates that the colors obtained from the reflectance spectrum closely corresponded to the colors representing the observed values. Consequently, utilizing the previously indicated approach enables the transformation of an WLI image obtained from an endoscope into an HSI image. The simulated data were employed to undertake a preliminary evaluation of the system’s performance. The spectrum emissions of the LEDs were examined during characterization, considering the sensitivities specified by the camera makers. The spectral curves of the 24-color Macbeth color checker chart were utilized for the training and validation datasets of the samples.

Although a methodology for converting a WLI picture to an HSI image, complete with reflectance data, has been established, it is necessary to simulate an NBI image for a conventional endoscope, which must be built based on band selection to identify distinct cancer kinds. Nonetheless, the Olympus endoscope features a reference NBI capture mode for algorithm comparison. Consequently, the NBI color calibration is performed based on the Olympus endoscope. Prior to this, the simulated NBI picture generated by the HSI conversion algorithm must closely resemble the authentic NBI image obtained from the Olympus endoscope. The usual 24-color checker has been utilized for this calibration as well. The NBI picture generated by the HSI conversion algorithm is juxtaposed with the authentic NBI image obtained from the Olympus endoscope. The CIEDE 2000 color disparity among the 24 color blocks is quantified and reduced. Post-correction, the mean color difference among the 24 color blocks was determined to be at 2.79, which is insignificant. After achieving color congruence between the simulated NBI, referred to as SAVE, and the actual NBI of the Olympus endoscope, the NBI image for the capsule endoscope requires further refinement. Three elements will influence the color disparity between authentic NBI and generated SAVE photos. These elements pertain to the light spectrum, the color-matching function, and the reflection spectrum. The initial observation revealed the CIEDE 2000 color disparity between WLI pictures from the capsule endoscope and the Olympus endoscope. The Olympus endoscope and the VCE have both been provided with the identical 24-standard color checker. A significant disparity arises between the two endoscopes due to the pronounced variance in their illumination spectra. Despite comparable strength at certain wavelengths, there exists a significant disparity in the band throughout the 450–540 nm range. As this is the zone where hemoglobin exhibits maximal light absorption, the illumination spectrum requires calibration. The Cauchy–Lorentz distribution was employed, as seen in Equation (5).

(5) $$f\left(x;x_{0},\gamma \right)=\frac{1}{\pi \gamma \left[1+{\left(\frac{x-x_{0}}{\gamma }\right)}^{2}\right]}=\frac{1}{\pi }\left[\frac{\gamma }{{\left(x-x_{0}\right)}^{2}+\gamma^{2}}\right]$$

**Figure 2:**
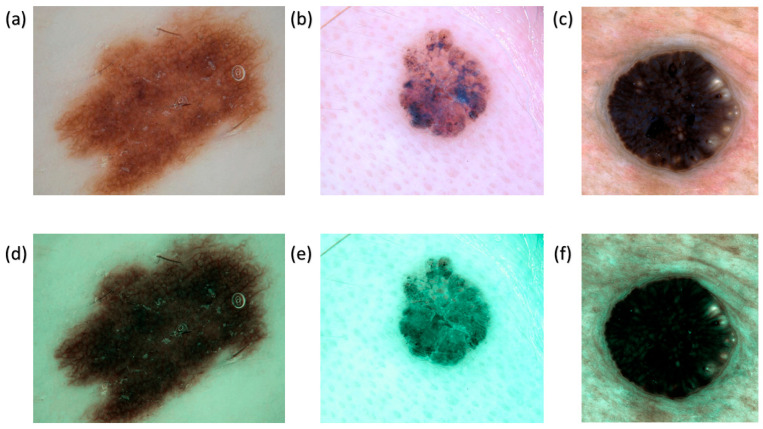
Examples of RGB and SAVE images. (**a**–**c**) show the AK, BCC and SK in WLI, while (**d**–**f**) show corresponding images of the AK, BCC and SK in SAVE.

The dual annealing optimization function is employed to optimize the illumination spectrum. This stochastic strategy was developed from an extended simulated annealing algorithm that integrates simplified classical simulated annealing (CSA) and fast simulated annealing (FSA), in conjunction with a method for conducting a local search based solely on certain parameters. The mean CIEDE 2000 color difference among the 24 colors is 5.36, which is insignificant. Although the peak absorption wavelengths of hemoglobin are 415 and 540 nm, the actual NBI picture obtained from the Olympus endoscope exhibits not only green and blue hues but also shades of brown, corresponding to a wavelength of 650 nm. Consequently, one may assert that there exists a nuanced picture post-processing that enhances the realism of the NBI films. Therefore, this study includes three more wavelength ranges at 600, 700, and 780 nm, alongside 415 and 540 nm. Figure 2 are some examples of converting the RGB images into corresponding SAVE images.

### 2.3. ML Algorithms

This study selected ten machine learning and deep learning models to thoroughly assess the classification of skin lesions. The selection comprises classical machine learning methods, including SVM with diverse kernel classifiers and RF, both of which are esteemed for their robustness, interpretability, and efficacy in medical image analysis. Furthermore, sophisticated deep learning architectures such as CNN, ResNet50, MobileNetV2, and YOLOv8 were utilized for their established capacity to autonomously extract hierarchical features and attain high precision in intricate image classification problems. This varied selection facilitates a comprehensive comparison between conventional and contemporary methodologies, guaranteeing an equitable evaluation of model efficacy in distinguishing AK, BCC, and SK. The selected models together encompass a wide range of techniques, offering insights into their relevance and efficacy for skin cancer diagnosis.

#### 2.3.1. Convolutional Neural Networks (CNNs)

##### Standard CNN

A CNN is one of the most representative neural networks in the area of deep learning. Computer vision based on CNN enables people to accomplish what had been considered impossible in the past few centuries, such as face recognition, autonomous vehicles, self-service supermarkets, and intelligent medical treatment [27]. The most common basic architecture of CNN consists of convolutional layers, pooling layers, nonlinear activation layers, and a fully connected layer. Normally, an image goes into the network through preprocessing via the input layer. Appropriate numbers of alternately assembled convolutional and pooling layers then process it and classify it by the fully connected layer [28]. The rectified linear unit RELU is a simple identity function for the positive input and zero for negative input given by Equation (6) [29].

(6) $$ReLU\left(x\right)=\max\left(0,x\right)=\left\{\begin{array}{c} x,\;\;if\;x\geq 0\\ \;0,\;\;otherwise \end{array}\right.$$

The Root Mean Square Error (RMSE) is the square root of the mean squared error (MSE) defined in Equation (7), where y is the true value, $\hat{y}\;is$ the predicted value, and *n* is the number of samples [30].

(7) $$RMSE=\sqrt{\frac{1}{n}\sum_{i=1}^{n}{\left(y_{i}-{\hat{y}}_{i}\right)}^{2}}$$

##### ResNet50

It introduces the Residual network, or ResNet, a new concept that benefits the solving of complicated tasks and increases the accuracy of detection. ResNet attempts to solve the difficulties in the training process of deep CNNs: saturation and degradation of accuracy. ResNet50 has 50 layers of residual networks [31]. It benefits from using skip links in any layer that degrade the design performance, which are skipped by regularization. Training a very deep neural network is therefore not hampered by vanishing gradients as a conventional CNN model. Like in the LSTM networks, parametric gates are used in these skip connections. These gates control the quantity of data crossing the skip connection. ResNet addresses the problem of a vanishing gradient and feature map vanishing during the training of enormous amounts of deep CNN. Since an identity link between non-adjacent layers does not affect the ideal mapping which the application task wants to finally produce, the ResNet works. This is because, with the identity connection, gradients have more of a chance for an additional shortcut channel, which allows for easier back propagation around [32]. For instance, the ResNet-50, during image classification tasks, resorts to cross-entropy loss. It is a measure of a predicted probability distribution against the actual distribution, which is the ground truth, as shown in Equation (8) [33].

(8) L=−∑i=1cyilog(yi^)

##### MobileNetV2

The MobileNetV2 model was proposed for image classification with an emphasis on the model’s portability. The main structure is based on its previous version, namely, MobileNetVl. The MobileNetV2 applies DSC for portability and improves the problem of destroying information in nonlinear layers in convolution blocks not only by using linear bottlenecks [34]. Both MobileNetV1 and MobileNetV2 take input images with a size of 224 × 224 × 3 pixels. Thus, the input images on the dataset are resized and cropped into 224 × 224 pixels. On the one hand, MobileNetV2 inserts 19 inverted residual bottleneck layers after the first convolution layer with 32_filters, and then the network ends with a pointwise convolution that produces an output with a size of 7 × 7 × 1280 pixels [35]. In the process of classification, MobileNetV2 is often combined with cross-entropy loss. Cross-entropy loss measures the difference between the probability distribution predicted by the model and the actual distribution (which usually corresponds to the ground truth labels) [36].

#### 2.3.2. Random Forest

RF has lower computational complexity and higher interpretability compared to other deep learning models. In the most recent research works, it is shown that RF is a relatively easy to implement method which can handle learning tasks with a small amount of training dataset yet demonstrates competitive results with CNNs. RF is another ensemble method where the RFs, with considerably different structures of trees and splitting variables, will introduce different instances of overfitting and outliers among several models in the tree ensembles. Therefore, it is voting in the final prediction that mitigates overfitting in case of a classification problem, while averaging solves the problem in regression problems [37]. Machine learning algorithms are used to develop diagnostic models for many diseases and they help the systems to learn the diagnosis data, identify useful patterns during the learning process, and minimize human interference to make decisions. The Gini index algorithm has multivariate feature importance scores which are quite inexpensive to compute, and the methods have been applied with success to high-dimensionality datasets arising from microarrays. When applying RF based on classification data, often Gini index needs to be used as shown in Equation (9) in order to decide on an approach to nodes on a decision tree branch where *P_i_* stands for the probability [38].

(9) $$Gini=1-\;\sum_{i=1}^{c}{\left(p_{i}\right)}^{2}$$

#### 2.3.3. YOLOv8

In the proposed system, the images are provided as an input at an identical size of 448 × 448 × 3 pixels and are taken through the powerful framework known as DarkNet, which comprises a series of convolutional layers designed to capture abstract features for the detection of objects. Further processing flattens it and takes it through a series of fully connected layers, finally resulting in a 7 × 7 grid [39]. Feature maps in YOLOv8 are divided into five types of scale features in descending order, which, for simplicity, can be represented as B1–B5, P3–P5, and N4–N5 in the backbone, FPN, and PAN structures, respectively. The original YOLOv8 employed the PAN-FPN structure, complementary to traditional FPN, hence employing a top–down form in transferring deep semantic features. By incorporating the B3–P3 and B4–P4, the feature pyramid is semantically enriched at the expense of certain loss of positioning information. PAN-FPN supplements the bottom–up structure behind FPN and adopts the image fusion of P4–N4 and P5–N5 to reinforce the learning of localization characteristic for achieving the complementary effect [40].

#### 2.3.4. Support Vector Machine

An SVM is one of the classical machine learning techniques that can still help in solving big data classification problems. SVMs differ in their basis on the complexity of the hypothesis space and empirical error measure of how well the model fits the training data. However, after the identification of model parameters, an SVM relies solely on a subset of these training instances, known as support vectors, for any future prediction. By definition, support vectors define the margins of the hyperplanes. Especially, big data environment multi-domain applications can be helped by it. However, an SVM is mathematically complex and computationally expensive [41]. The main idea of the SVM is to estimate a model in which we should determine the best hyper-plane that is able to separate the data. The hyperplane is mathematically expressed in Equation (10), where the *w* is the weight vector, and *x* is the input by the bias value [42].

(10) $$w^{T}\cdot \;x=-b$$

#### 2.3.5. Logistic Regression

LR is a standard probabilistic statistical classification model that has had great use across disciplines. Unlike linear regression, the outcome of the LR on one sample is a probability that is positive or negative, and this probability depends on one linear measure of the sample. Hence, LR is widely used in classification. LR characterizes the relevance or appropriateness of an independent or predictor variable through the size of the coefficient and equally discloses the tendency in the direction of their relationship or association as positive or negative. LR is a kind of regression that is used to predict a dichotomous dependent variable. In constructing the equation for the LR, the maximum-likelihood ratio was utilized in deriving the value of the variables to know which among them is statistically significant. LR can be applied to predict the presence or absence of a certain characteristic or outcome based on the values of a set of predictor variables [43]. Also, LR is said to be discriminatory since the algorithm is actually learning how it should distinguish these classes from each other. The logistic function, also logit or the sigmoid function, is to force the result from the cost function to be a probability output between 0 and 1, which is shown in Equation (11) [44].

(11) $$h\left(t\right)=\frac{1}{1+e^{-t}}$$

#### 2.3.6. Support Vector Machine (SVM)

##### SVM—SGD Classifier

SGD is also referred to as incremental gradient descent; it is defined as an iterative method for optimizing a differentiable objective function—a kind of stochastic approximation of the gradient descent optimization [45]. SGD is a variant of GD that concerns itself with random probability, and it is stochastic, such that at each and every iteration, only one sample is selected for training the model [46]. This loss function, when selected for classification problems, is defined as the sum of squares of all output values, as shown in Equation (12) [18].

(12) L=1N ∑i=1Nmax(0,1 − yi⋅ yi^)

##### SVM—LOG Classifier

It is a kind of classifier which enables the SVM to carry out a two-dimensional (2-D) classification for data that were once one-dimensional. Normally, a kernel function can project low-dimensional-space data into higher-dimensional-space data, as shown in Equation (13) [47].

(13) ⟨x1⋅x2⟩←K(x1,x2)=⟨Φ(x1)⋅Φ(x2)⟩

The linear kernel function is commonly described as shown in Equation (14).

(14) K(x,xj)=x⋅xT

##### SVM—Polynomial Classifier

It is a kind of SVM where the data transformations carried out on the input are by done so polynomial functions into a higher dimensional space. The polynomial kernel function uses the dot product between the input data points and adds the constant to the result, raised to a power specified by the degree parameter of the function. The result of this transformation is to give a new set of features that represent non-linear relationships between the input data [48]. Another aspect of the polynomial kernel function is that it is directional; this means the output will be in a specific direction, depending on the two vectors’ direction in a low dimensional space. This becomes evident because of the dot product involved in the kernel. The magnitude of the output will also be dependent on the magnitude of vector $x_{i}$, as shown in Equation (14) [47].

## 3. Results

**Figure 3:**
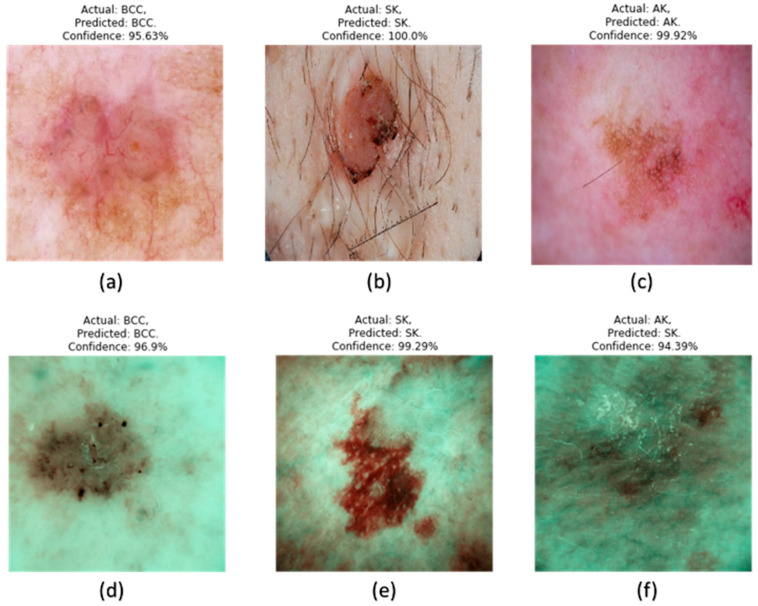
Examples of the prediction of skin cancer results in RGB imaging and SAVE. (**a**–**c**) show the results of BCC, SK and AK with confidence results of 95.63%, 100% and 99.92%, respectively, in RGB imaging, while (**d**–**f**) show BCC, SK and AK with confidence results of 96.9%, 99.29% and 94.39%, respectively, in SAVE imaging.

In this study, various machine learning models regarding skin cancer classification were used: AK, BCC, and SK in WLI and SAVE systems, as shown in Figure 3. For each of the models, the key evaluation metrics include precision, recall, F1-score, and accuracy as shown in Table 1. CNNs performed excellently well—especially when combined with the SAVE system. Combined with WLI, these reached an accuracy of 94%, while for AK, precision was slightly lower than recall, at 91%. Nevertheless, the addition of the SAVE system resulted in a great increase, placing it up to 98% in accuracy, with perfect precision of 100% for SK and almost perfect recall for all classes. This indicated that CNN, working on spectral data, managed to classify skin cancer.

The random forest models also led to good results, especially when using SAVE (see Supplementary Materials Figure S4 for the confusion matrix of RF. (a) RGB imaging and (b) SAVE). With WLI, the accuracy of RF achieved 90.62%, but precision with respect to BCC was not very good, at 87%, and recall was better at 95%. Global improvement, achieved with SAVE, increased accuracy to 92.19%. Both precision and recall for all classes increased, and especially for BCC, it reached 91%. This pointed out that spectral information played an important role in model performance improvement.

The YOLOv8 results, when designed for live detection, were mixed. The model using WLI reached 84% accuracy, with the precision and recall for SK both at 78% and 87%, respectively. SAVE increased the precision for AK to 92% but also decreased the recall to 80%. This suggests that although SAVE helps with the precision of YOLOv8, it is not able to guarantee high recall among all types of skin cancer.

Support vector machines (SVMs) showed a varied performance according to the applied classifier (see Supplementary Materials Figure S5 for the confusion matrix of SVM-Log. (a) RGB imaging and (b) SAVE). The linear SVM with WLI showed relatively poor results: its AK recall was only 50%, which lifted the general accuracy to 71%. SAVE slightly improved the performance, especially for SK, where recall increased to 86%; however, the SVM remained far behind in this task compared to more advanced models like CNN and RF.

ResNet50 had poor accuracy with WLI and provided an overall accuracy of 71% (see Supplementary Materials Figure S3 for the confusion matrix of Resnet v50. (a) RGB imaging and (b) SAVE. S4). Again, the precision for AK was very low at 60%, which shows it did not manage to predict this cancer well. However, the addition of SAVE enhanced the accuracy up to 76%, with important increases in terms of precision and recall for SK, up to 68% and 89%, respectively. It seems that ResNet50 can benefit from spectral data but is worse than other deep learning models.

MobileNetV2, being effective in low-resource settings, attained 69% accuracy with WLI (see Supplementary Materials Figure S2 for the Confusion matrix of Mobilnetv2. (a) RGB imaging and (b) SAVE). It performed badly in the detection of SK, with low precision at 60%. This was significantly corrected by SAVE, improving the overall accuracy to 74%, with SK detection increasing significantly to 82% and 75% for precision and recall. This explains that although MobileNetV2 gains from spectral information, it is less robust in comparison to CNN and RF.

Logistic regression: It is an inherently simpler model, and so it performed well but not particularly great, especially with SAVE (see Supplementary Materials Figure S1 for the Confusion matrix of Logistic Regression. (a) RGB imaging and (b) SAVE). Its score for WLI came in at 89.84% with balanced precision–recall for all categories. For SAVE, it increased it up to 91.41%, classifying the precision and recall for SK both over 90%. So, even quite basic models can obtain a gigantic lift from spectral data.

On the other hand, using SVM with a stochastic gradient descent (SGD) classifier did not work well with WLI, with only 45% accuracy obtained and a recall of 21% for AK (see Supplementary Materials Figure S6 for the confusion matrix of SVM-SGD. (a) RGB imaging and (b) SAVE). However, in conjunction with SAVE, the performance of the model was considerably better, with an 82% overall accuracy, while the balance between precision and recall across all classes significantly improved, especially for AK, which increased to 77% in terms of precision.

The Logistic Classifier, as an SVM variant, also seemed to find WLI extremely difficult and eventually achieved just 49% accuracy (see Supplementary Materials Figure S7 for the confusion matrix of SVM-Polynomial. (a) RGB imaging and (b) SAVE). Performance shot up to 82% with SAVE, and its precision and recall for AK were increased significantly to 83% and 93%, respectively. This means that, although this SVM variant performed quite badly on WLI, it can gain quite a bit from the spectral enhancement that SAVE brought in.

Among the SVM models, the Polynomial Classifier with this SVM model demonstrated very strong performance (see Supplementary Materials Figure S8 for the confusion matrix of SVM. (a) RGB imaging and (b) SAVE). WLI combined with the model showed 88% accuracy, giving high values of precision and recall for BCC and SK of 89% and 95%, respectively. Further enhancement in its performance by SAVE raised the performance to 91%, with high values of both precision and recall on all cancer types, especially on SK, where both rates were over 90%, which proved the effectiveness of the polynomial classifier with spectral data.

## 4. Discussion

One of the key strengths of this study is its use of the SAVE algorithm, which is designed to augment multiple machine learning models with more granular spectral information. Such an approach will allow a model to sense the subtle differences between the tissue characteristics that might not be very vivid through conventional WLI imaging. Captured HSI data give a higher precision to the classification of cancerous and non-cancerous tissues, which significantly impacts diagnostic accuracy. Notwithstanding, this paper presents a few weaknesses. Firstly, the sample size that was used for training and testing the models was quite small; thus, the generalizability of the results may be hampered. Although the many models worked fine on the given dataset, how well it worked for a larger and more diverse set of skin images—usually with many ethnicities and diverse skin types—is indeterminate. This highlights the need for validation of the model with larger datasets to make it stronger and more relevant to real clinical settings. Secondly, in this study, the SAVE conversion tool was used as a basic tool, insofar as it is effective; however, this kind of technology is resource-consuming at the present time. Cost and complexity are among the other main issues with the module that could hinder its widespread use, especially within resource-constrained healthcare environments. The results of this study are promising, and further research with less expensive alternatives, such as multispectral imaging, can be carried out without decreasing the level of diagnostic accuracy. The SAVE conversion algorithm processes each skin image in approximately 0.8–1.2 s on a standard NVIDIA GTX 1080 graphics card. The conversion of hyperspectral spatial data for input into NBI, along with the classification network, is fully parallelizable, enabling the processing of hundreds of images within an hour through batch processing. Additionally, per-image latency could be significantly reduced to below 1 s by employing optimized inference engines or edge-AI accelerators. Recently developed handheld, portable hyperspectral cameras and integrated AI computing hardware have enabled the integration of SAVE into routine dermatological practice, facilitating real-time lesion analysis and high-throughput screening at both clinical and community levels. Furthermore, this study did not have an extensive exploration of the false-positive and false-negative rates among different cancer types, which is very important for consideration as a part of the estimation of clinical utility of the model in diverse diagnostic situations. Prior modification should also be carried out in order to extend the dataset to include more skin tones, types of cancer, and imaging conditions to make the model more broadly applicable in clinical fields. Integration of real-world clinical data could make the model adjust better to the complex variabilities in practical medical environments. Future works should investigate integrating SAVE into other machine learning models in order to boost classification accuracy further. Future work needs to further consider the possibilities for improving the ability of such models to perform real-time detection, which should be particularly useful in clinical settings where accurate diagnosis is important. This study utilized many established machine learning and deep learning architectures in their default configurations to deliver a thorough baseline assessment of the SAVE-enhanced imaging method. This decision was driven by the intention to uphold simplicity and reproducibility while evaluating the core efficacy of our strategy across several models. While contemporary and advanced architectures—such as transformer-based models or convolutional networks enhanced with attention mechanisms like SE blocks—have shown superior performance in numerous image classification tasks, the incorporation of these techniques was not within the parameters of this preliminary study [49]. We recognize that implementing these modifications could enhance classification accuracy and resilience. Future endeavors will concentrate on integrating these cutting-edge architectural enhancements to expand upon the encouraging results demonstrated herein and investigate their effects within the realm of hyperspectral skin lesion classification.

## 5. Conclusions

This study introduces a novel Spectrum-Aided Vision Enhancer (SAVE) algorithm, which effectively converts RGB images into narrow-band imaging (NBI) images by selecting specific narrow bands to enhance the contrast of cancerous lesions. The application of SAVE significantly improves the detection of skin cancer compared to traditional RGB imaging methods. Among the machine learning models tested, convolutional neural networks (CNNs) demonstrated the highest accuracy, reaching 98% with SAVE, an improvement of 4% from RGB images. The enhanced spectral information provided by SAVE allows for more precise differentiation between cancerous and non-cancerous tissues, leading to improved diagnostic accuracy. However, this study acknowledges its limitations, including the small sample size, which may affect the generalizability of results across diverse populations and skin types. Additionally, the complexity and cost of SAVE technology could pose challenges for widespread adoption in resource-constrained healthcare settings. Future research should focus on expanding the dataset to include more diverse skin tones and cancer types, as well as exploring cost-effective alternatives like multispectral imaging. Integrating real-world clinical data could further refine the model’s accuracy and applicability in practical medical environments. Investigating the potential of SAVE in conjunction with other machine learning models may also enhance classification accuracy. Emphasizing real-time detection capabilities could be particularly beneficial in clinical settings where timely and accurate diagnosis is crucial. This study is constrained by a modest sample size and insufficient demographic data, potentially impacting the generalizability of the findings. Moreover, the intricacy and expense of hyperspectral imaging, together with reliance on standard model configurations, may hinder clinical implementation and optimal efficacy, respectively.

## Figures and Tables

**Table 1:** The overall results of all the 10 models.

Framework	Model	Type	Metrics			
			Precision	Recall	F1-Score	Accuracy
CNN	WLI	AK	91.29%	96.15%	94.56%	94%
		BCC	96.78%	91.52%	93.28%	
		SK	96.63%	96.44%	96.68%	
	SAVE	AK	95.75%	100.00%	98.02%	98%
		BCC	98.52%	98.43%	98.85%	
		SK	100.00%	97.75%	98.69%	
RF	WLI	AK	87.36%	94.36%	91.64%	90%
		BCC	87.42%	95.28%	91.15%	
		SK	98.91%	84.49%	90.74%	
	SAVE	AK	90.25%	93.75%	92.45%	92%
		BCC	91.71%	89.00%	90.52%	
		SK	95.62%	95.42%	95.45%	
YOLOv8	WLI	AK	83.95%	88.67%	85.48%	84%
		BCC	89.51%	78.78%	83.26%	
		SK	78.38%	87.73%	82.78%	
	SAVE	AK	92.63%	80.64%	86.14%	85%
		BCC	75.45%	89.37%	82.53%	
		SK	87.37%	87.42%	87.28%	
SVM	WLI	AK	73.00%	50.34%	59.96%	71%
		BCC	67.52%	72.96%	70.61%	
		SK	73.75%	83.42%	78.24%	
	SAVE	AK	67.46%	65.25%	66.24%	73%
		BCC	78.34%	65.72%	70.04%	
		SK	72.69%	86.53%	79.63%	
ResNet50	WLI	AK	60.81%	70.77%	65.89%	71%
		BCC	84.47%	63.00%	72.47%	
		SK	70.29%	81.27%	75.63%	
	SAVE	AK	76.24%	74.54%	75.24%	76%
		BCC	85.37%	67.49%	75.75%	
		SK	68.59%	89.37%	77.54%	
MobileNetV2	WLI	AK	84.11%	50.74%	63.65%	69%
		BCC	75.86%	70.91%	73.08%	
		SK	60.61%	84.72%	70.96%	
	SAVE	AK	68.99%	63.31%	65.85%	74%
		BCC	72.17%	82.67%	76.21%	
		SK	82.36%	75.88%	78.32%	
Logistic Regression	WLI	AK	87.89%	91.12%	89.37%	89%
		BCC	88.37%	93.38%	90.45%	
		SK	95.52%	86.14%	90.20%	
	SAVE	AK	89.99%	93.29%	91.91%	91%
		BCC	72.71%	82.11%	76.38%	
		SK	92.35%	90.78%	91.81%	
SVM- SGD Classifier	WLI	AK	100.00%	21.39%	35.63%	45%
		BCC	83.27%	12.72%	21.98%	
		SK	39.33%	100.00%	56.93%	
	SAVE	AK	77.35%	95.96%	85.52%	82%
		BCC	82.42%	70.63%	75.37%	
		SK	88.33%	84.78%	86.61%	
SVM- LOG Classifier	WLI	AK	90.78%	24.56%	38.83%	49%
		BCC	41.42%	100.00%	58.58%	
		SK	100.56%	22.75%	36.93%	
	SAVE	AK	83.96%	93.22%	88.64%	82%
		BCC	81.73%	65.67%	72.46%	
		SK	82.45%	87.85%	84.73%	
SVM- Polynomial Classifier	WLI	AK	90.23%	83.30%	86.31%	88%
		BCC	89.75%	89.55%	89.88%	
		SK	86.00%	95.64%	90.27%	
	SAVE	AK	91.75%	83.95%	87.09%	91%
		BCC	91.23%	93.78%	92.78%	
		SK	92.65%	96.53%	94.65%	

## Data Availability

Data underlying the results presented in this paper are not publicly available at this time but may be obtained from the authors upon reasonable request.

## Supplementary Materials

The following supporting information can be downloaded at: https://www.mdpi.com/article/10.3390/bioengineering12070755/s1, Figure S1. Confusion matrix of Logistic Regression. (a) RGB imaging and (b) SAVE. Figure S2. Confusion matrix of Mobilnetv2. (a) RGB imaging and (b) SAVE. Figure S3. Confusion matrix of Resnet v50. (a) RGB imaging and (b) SAVE. Figure S4. Confusion matrix of RF. (a) RGB imaging and (b) SAVE. Figure S5. Confusion matrix of SVM-Log. (a) RGB imaging and (b) SAVE. Figure S6. Confusion matrix of SVM-SGD. (a) RGB imaging and (b) SAVE. Figure S7. Confusion matrix of SVM-Polynomial. (a) RGB imaging and (b) SAVE. Figure S8. Confusion matrix of SVM. (a) RGB imaging and (b) SAVE. Ref. [50] is cited in Supplementary Materials.

## Abbreviations

AK: Actinic Keratosis; BCC: Basal Cell Carcinoma; CNN: Convolutional Neural Network; EC: Esophageal Cancer; HSI: Hyperspectral Imaging; ISIC: International Skin Imaging Collaboration; LED: Light Emitting Diode; LR: Logistic Regression; MM: Malignant Melanoma; MRI: Magnetic Resonance Imaging; mAP: Mean Average Precision; NBI: Narrow Band Imaging; NMSC: Non-Melanoma Skin Cancer; PC: Principal Component; PCA: Principal Component Analysis; RF: Random Forest; ReLU: Rectified Linear Unit; RMSE: Root Mean Square Error; ROI: Region of Interest; RT-DETR: Real-Time Detection Transformer; SAVE: Spectrum-Aided Vision Enhancer; SCC: Squamous Cell Carcinoma; SGD: Stochastic Gradient Descent; SVM: Support Vector Machine; VCE: Video Capsule Endoscope; WLI: White Light Imaging; YOLO: You Only Look Once
