# Supplementary material for: Hyperspectral Imaging for Enhanced Skin Cancer Classification Using Machine Learning
Source: Bioengineering (Basel). 2025 Jul 11;12(7):755. doi: 10.3390/bioengineering12070755 (PMC12292285; doi:10.3390/bioengineering12070755)
Supplement: Supplementary file 1 [file bioengineering-12-00755-s001.zip › bioengineering-3668891-supplementary.pdf]

Hyperspectral Imaging for Enhanced Skin Cancer Classification Using Machine Learning: Supplementary Materials

Evaluation Indices:

Evaluation metrics are quantitative, mathematical, and objective measures for grading the accuracy, performance, or the efficacy of statistical or machine learning algorithms[1]. These yield crucial information pertaining to understanding the performance of the model and can help in the comparison of different models, returns or the very same model, with the same algorithm configurations. Accuracy measures the percentage of samples in the data set that were correctly categorized by the model as positive, thus measures the capacity of the model to avoid the false positives.

$$\text{Accuracy} = \frac{TP+TN}{TP+TN+FP+FN} \quad \text{(S1)}$$

Recall computes the ratio of correct positive cases relative to all the cases that are positive actually; it deals with the capacity of the model to diagnose all instances of a particular class.

$$\text{Precision} = \frac{tp}{tp+fp}$$
$$\text{Recall} = \frac{tp}{tp+fn} \quad \text{(S2)}$$

The F1 Score, which is formed by the precision score and the recall score, and summing them. The F-1 Score, therefore, delivers a more accurate account of the performance of the model in the balanced mode either of false positives or false negatives.

$$F = 2 \cdot \frac{\text{precision} \cdot \text{recall}}{\text{precision} + \text{recall}} \quad \text{(S3)}$$

Recall values range from 0 to 1 and the average precision (AP) means have been computed on average precision values. mAP formula is based on the following sub metrics: Confusion matrix,Intersection over Union (IoU), Miss rate ,Hit rate. Four characteristics are necessary for developing a confusion matrix which is True Positive (TP) ,True Negatives (TN), True Negative (TN) and False Negatives (FN) .mAP50 is interpreted as the Mean average precision which is calculated at intersection over union (IoU) of 0. 50. It relates to how well the model performs based on the subset of detections that every algorithm should ideally be able to handle easily .mAP50-95 is the mean average precision at the intersection of union of different levels of IoU thresholds from 0.50 to 0.95. Thus, it provides an overview of the model's performance depending on how difficult it is to find an object in an image.

$$\text{mAP} = \frac{1}{N}\sum_{i=1}^{N} \text{AP}_i \quad \text{(S4)}$$

Result

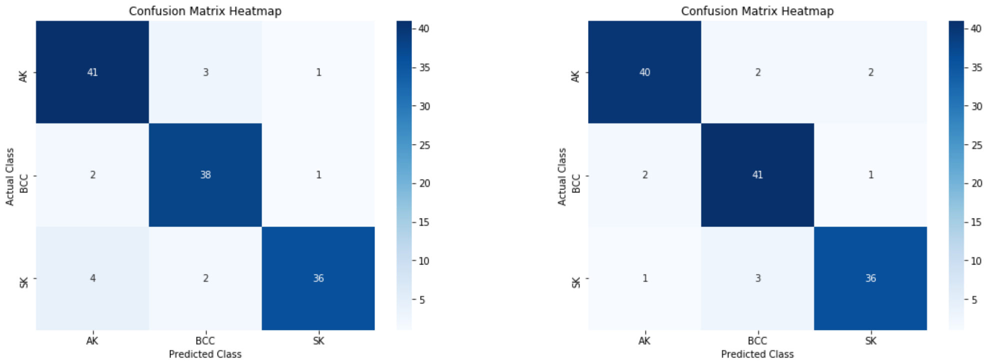

(a) (b)

Figure S1. Confusion Matrix of Logistic Regression. (a) RGB Imaging and (b) SAVE

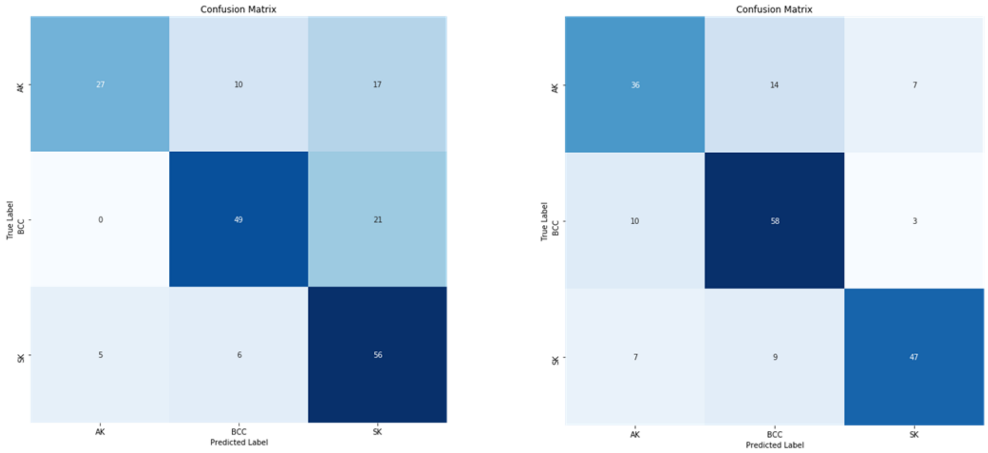

(a) (b)

Figure S2. Confusion Matrix of Mobilnetv2. (a) RGB Imaging and (b) SAVE

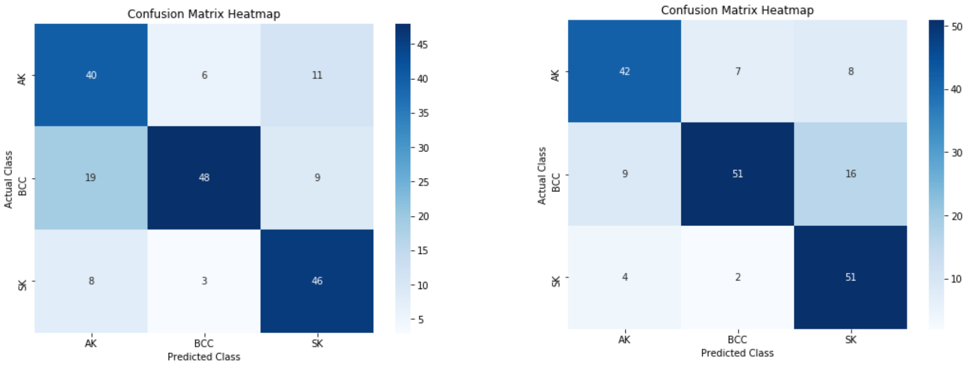

(a) (b)

Figure S3. Confusion Matrix of Resnet v50. (a) RGB Imaging and (b) SAVE

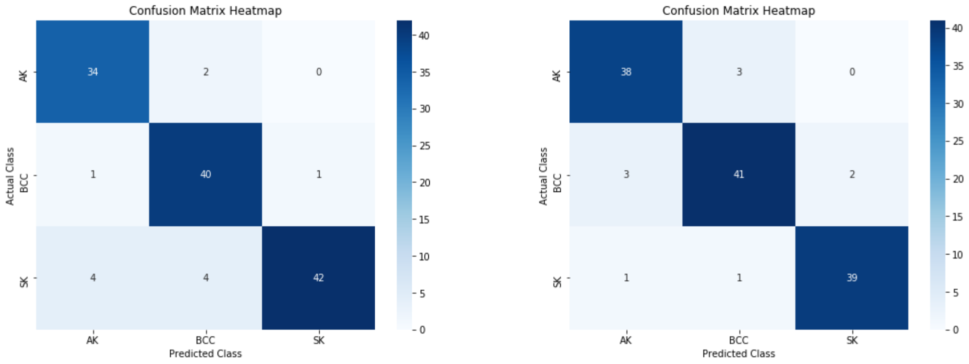

(a) (b)

Figure S4. Confusion Matrix of RF. (a) RGB Imaging and (b) SAVE

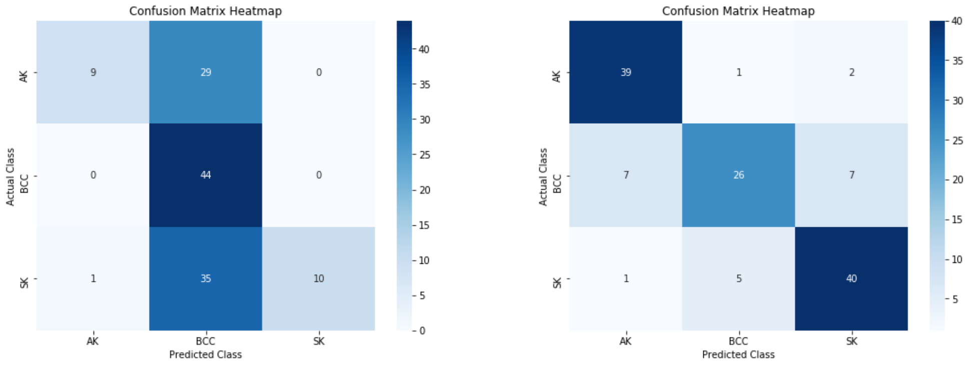

(a) (b)

Figure S5. Confusion Matrix of SVM- Log. (a) RGB Imaging and (b) SAVE

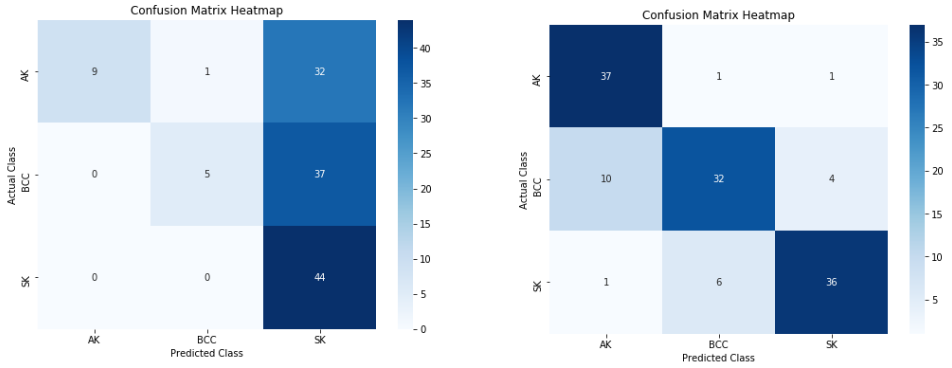

(a) (b)

Figure S6. Confusion Matrix of SVM-SGD. (a) RGB Imaging and (b) SAVE

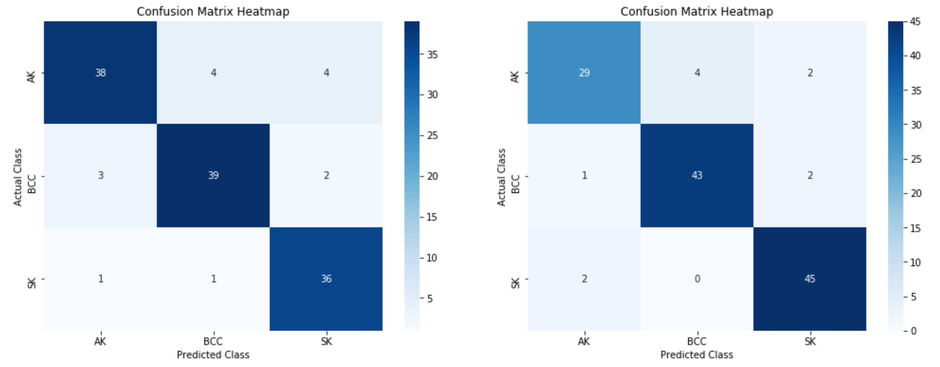

(a) (b)

Figure S7. Confusion Matrix of SVM-Polynomial. (a) RGB Imaging and (b) SAVE

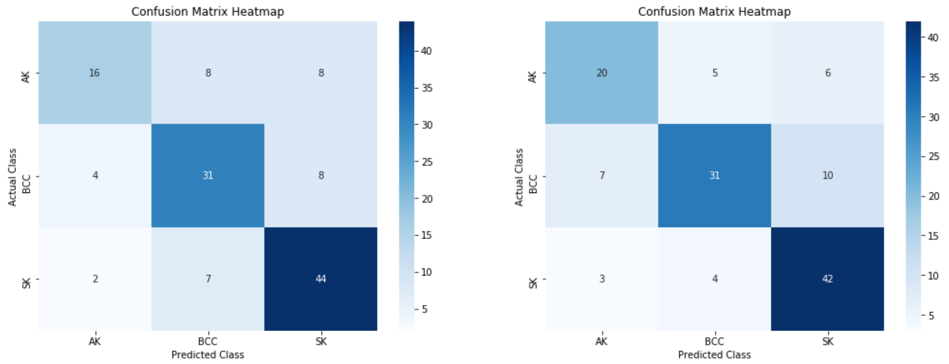

(a) (b)

Figure S8. Confusion Matrix of SVM. (a) RGB Imaging and (b) SAVE

1 Padilla, R., Netto, S. L. & Da Silva, E. A. in *2020 international conference on systems, signals and image processing (IWSSIP).* 237-242 (IEEE).
